# Antibody in Breastmilk Following Pertussis Vaccination in Three-time Windows in Pregnancy

**DOI:** 10.1097/INF.0000000000004696

**Published:** 2025-02-14

**Authors:** Olwenn Daniel, Myles Loughnan, Miranda Quenby, Krina Chawla, Vanessa Greening, Paul T. Heath, Christine E. Jones, Asma Khalil, Laxmee Ramkhelawon, Anna Calvert, Kirsty Le Doare

**Affiliations:** From the * Centre for Neonatal and Paediatric Infection and Vaccine Institute, School of Health & Medical Sciences, City St George’s, London, UK; † Department of General Medicine, The Royal Children’s Hospital, Melbourne, Australia; ‡ St George’s University Hospitals NHS Foundation Trust, London, United Kingdom; § NIHR Southampton Clinical Research Facility and Biomedical Research Centre, University Hospital Southampton NHS Foundation Trust, Southampton, United Kingdom; ¶ Faculty of Medicine and Institute for Life Sciences, University of Southampton, Southampton, United Kingdom; ∥ Makerere University-Johns Hopkins University Research Collaboration, Kampala, Uganda; ** Pathogen Immunology Group, UK Health Security Agency, Porton Down, Salisbury, United Kingdom.

**Keywords:** *Bordetella pertussis*, pertussis-containing vaccine, breastmilk, immunoglobulin A, maternal vaccine

## Abstract

**Background:**

Pertussis-containing vaccines are routinely offered in the UK at 16–32 weeks of gestation and have been shown to be safe and effective, but there remains debate about the best timing for vaccination. Most research into this has focused on serologic immunity, but breastmilk is also important in infant immunity, and the amount of IgA in breastmilk may impact mucosal immunity. It is important to understand if the timing of vaccination in pregnancy affects the concentration of IgA in breastmilk.

**Methods:**

Participants recruited as part of the MAMA (Maternal Antibody in Milk After Vaccination) and OpTIMUM (Optimizing the Timing of Whooping Cough Immunisations in Mums) trials received a pertussis-containing vaccine during pregnancy, either before 24 weeks, between 24 and 27^+6^ weeks or between 28 and 31^+6^ weeks. Samples of colostrum within 24 hours of delivery and breastmilk at 14 days were collected. Pertussis toxin, pertactin, tetanus toxoid and diphtheria toxoid specific-IgA levels were measured using a multiplex immunoassay.

**Results:**

There was no difference in specific IgA levels against pertussis toxin, pertactin, tetanus toxoid and diphtheria toxoid between the groups vaccinated within different time periods. For all antigens, there was decay in antigen-specific IgA levels between colostrum and breastmilk at 14 days.

**Conclusion:**

Our results suggest that the timing of administration of a pertussis-containing vaccine in pregnancy does not impact on antigen-specific IgA concentration in colostrum or breastmilk at 14 days.

Pertussis is a vaccine-preventable respiratory infection caused by the bacterium *Bordetella pertussis*. Infection can result in significant morbidity and mortality, primarily in infants less than 3 months of age born to mothers unvaccinated with a pertussis-containing vaccine in pregnancy and who have not yet received the benefits of their own vaccinations.^1,2^ Despite well-established vaccination programs against pertussis, there was an increase in the number of cases seen in many countries from 2005, associated with increased hospitalizations and deaths in young infants.^3^ In response to this, many high-income countries, including the United Kingdom,^4–6^ introduced antenatal pertussis vaccine programs that have been shown to be safe and effective.^7^

Recommendations about the timing of antenatal pertussis vaccination vary between countries, and there has been debate about the optimal timing of vaccination for the best serological protection of the infant. Two vaccine effectiveness studies have reported better protection after vaccination in the third rather than second trimester^8,9^ and a third has shown no impact of timing on effectiveness,^10^ whereas several immunogenicity studies have suggested that vaccination earlier in the third trimester results in higher antibody concentrations than vaccination later in the third trimester.^11–13^ Vaccination was initially recommended between 28 and 32 weeks gestation in the United Kingdom, but this advice was updated in 2016 to 16–32 weeks, and extending this time window has been shown to have advantages for increasing opportunities to vaccinate and for preterm infants.^14^

Although most research has focused on serological immunity, antenatal vaccination has been shown to increase antibody titers in breastmilk against pertussis. One study found an approximately 3-fold increase in the geometric mean concentration (GMC) of IgA to pertussis toxin (PT) in the breastmilk at 8–9 weeks postpartum of antenatally vaccinated women compared to women not vaccinated in the preceding 5 years.^15^ Another study found greater filamentous hemagglutinin-specific IgA in colostrum and breastmilk at 2 weeks, in Tdap-vaccinated women after 20 weeks of gestation than in unvaccinated women.^16^ A retrospective Belgian study of 234 participants found significantly higher PT-specific-IgG GMC in the colostrum of vaccinated women delivering at term compared with unvaccinated controls.^17^

While the exact mechanism of protection from antibodies in breastmilk is uncertain, increasing antibody titers in breastmilk plausibly increases protection against pertussis in the neonate through the enhancement of mucosal immunity. Using mice intracerebrally challenged with *B. pertussis,* a study has shown the effect of IgA contained in human colostrum on bacteria neutralization.^18^ PT-specific IgA and IgG from maternal breastmilk have also been shown to remain stable in the infant gastrointestinal tract during digestion.^19^ Increasing pertussis-specific IgA in breastmilk through maternal vaccination could plausibly increase protection against pertussis in the neonate, but the impact of timing of vaccination on pertussis antibody concentration has not previously been explored. In this analysis, we studied IgA concentrations in colostrum and breastmilk at 14 days, after vaccination either before 24 gestational weeks, between 24 and 27^+6^ gestational weeks, or between 28 and 31^+6^ gestational weeks in pregnancy.

## METHODS

The participants included in this analysis were recruited from 2 studies in the United Kingdom: Maternal Antibody in Milk After Vaccination (MAMA, NCT03982732) and Optimizing the Timing of Whooping Cough Immunisations in Mums (OpTIMUM, NCT03908164).

The MAMA study was a single-center observational study in which women were recruited postnatally. Participants were eligible if they had a singleton pregnancy, had received pertussis vaccination between 16 and 32 gestational weeks and were planning to breastfeed. Women were excluded if they had a known immunodeficiency. All participants had received a pertussis-containing vaccine as part of routine antenatal care, and the timing of vaccination was self-reported by participants, being checked with medical records if participants were uncertain. Participants were assigned to 3 groups according to the time at which they had received vaccination (<24 weeks, 24–27^+6^ weeks and 28–31^+6^ weeks). Participants provided a sample of colostrum within 48 hours of delivery and a breastmilk sample at 14 and 42 days.

The OpTIMUM study was an equivalence study in which participants were randomized into 3 gestational age groups (<24 weeks, 24–27^+6^ weeks and 28–32 weeks). Randomization was on a 1:1:1 ratio using a computerized block randomization list. Participants were eligible if they were pregnant and had not yet received pertussis vaccination, willing and able to comply with study procedures and provide informed consent and had received a 20-week anomaly scan with no life-limiting congenital anomalies identified. Exclusion criteria were maternal age of less than 16, confirmed or suspected pertussis in previous 5 years, known diagnosis of immune deficiency, receiving immunosuppressive medication within 6 months of enrollment, or in the opinion of the investigator was unlikely to complete follow-up. This was a multicenter study run in 6 sites. Participants in 2 sites (St. George’s, University of London and University Hospitals Southampton NHS Foundation Trust) were asked if they wished to take part in the breastmilk substudy. All participants in the OpTIMUM study had a cord blood sample taken at delivery and participants in the breastmilk substudy gave a sample of colostrum within 48 hours of delivery. A further breastmilk sample was collected at 14 and 42 days.

Due to the COVID lockdowns, the number of participants that attended the 42-day visit in the OpTIMUM study was very low (see Figure, Supplemental Digital Content 1, http://links.lww.com/INF/G3), and this time point was therefore excluded from further analysis.

Participants in both studies received Boostrix-inactivated poliovirus vaccine (GlaxoSmithKline; London, UK). Boostrix-IPV contains PT (8 μg), filamentous hemagglutinin (8 μg), pertactin (PRN) (2.5 μg), diphtheria toxoid (DT) (not less than 2 international unit), tetanus toxoid (TT) (not less than 20 international units) and inactivated poliovirus types 1–3 (type-1 40 D-antigen unit, type-2 8 D-antigen unit and type 3 32 D-antigen unit).

Ethical approval for the MAMA study was given by the West of Scotland Research Ethics Committee (18/WA/0171) and for the OpTIMUM study was given by the York and Humber Research Ethics Committee (19/YH/0050).

### Sample Processing

Colostrum and breastmilk samples were centrifuged first at 1000 g at 4 °C for 10 minutes and then at 10,000 g at 4 °C for 30 minutes to separate the lipid fraction from whey, which was then stored at −80 °C.

### IgA Measurement

An in-house Multiplex assay was used to measure antigen-specific colostrum and breastmilk IgA. MagPlex microspheres (Luminex DiaSorin, Italy) were conjugated to 4 antigens: PT, PRN, DT and TT (181, 187, 151 and 191B, respectively, List Biological Laboratories, Campbell, United-States).

Colostrum and breastmilk samples were diluted to 1:100, 1:500 and 1:2.500, next to a curve prepared from the Pertussis Antiserum World Health Organization International standard (1:60 dilution and 2-fold serial dilution, NIBSC 06/140, UK). Samples and microspheres were incubated overnight at 300 rpm and 2–8 °C, and R-phycoerythrin-conjugated goat antihuman IgA secondary antibody (1:200, 50 µl/well, 109-115-011, Jackson ImmunoResearch, Ely, UK) was added for 1 hour 30 minutes at 300 rpm at room temperature. Plates were read with Bio-Plex 200 (Bio-Rad, Hercules, CA).

### Statistical Analysis

Mean fluorescence intensity was interpolated into concentrations (arbitrary units) with Bio-Plex Manager Software 6.2. IgA results under the assay limits of quantification were attributed to 0.001 AU. On GraphPad Prism 10.2.2, the GMC levels of IgA were plotted. Because of the impact of the COVID-19 pandemic on the collection of the final breastmilk samples we included only the timepoints of colostrum at delivery and breastmilk at 14 days.

## RESULTS

This analysis was performed on samples collected from 2 studies: MAMA and OPTIMUM (see Figure, Supplemental Digital Content 1, http://links.lww.com/INF/G3). There were 104 participants with either a colostrum sample or a breastmilk sample at 14 days, or a sample at both time points (43 MAMA, 61 OpTIMUM). These are included in the analysis.

Overall, there were more participants vaccinated at <24 gestational weeks (GW) (n = 41) and 24–27^+6^ GW (n = 40) than at 28–31^+6^ GW (n = 23). Demographic details are described in Table, Supplemental Digital Content 2, http://links.lww.com/INF/G4.

### IgA in Colostrum and Breastmilk

IgA concentrations against any of the tested antigens between women vaccinated in either of the 3 gestational age groups were similar for colostrum (see Figure, Supplemental Digital Content 3, http://links.lww.com/INF/G5) and breastmilk at 14 days (see Figure, Supplemental Digital Content 4, http://links.lww.com/INF/G6).

Colostrum PT and PRN-specific-IgA are compared to cord serum PT and PRN-specific-IgG collected in the OpTIMUM study in Figure, Supplemental Digital Content 5, http://links.lww.com/INF/G7 and no correlation was found.

### Antibody Decay

Antibody concentrations were greater in colostrum than in breastmilk collected at 14 days, with IgA decay between these 2 time points. The decay percentages were similar between study groups (PT : percent decay <24 GW = 99.89%, 24–27^+6^ GW = 99.98%, 28–31^+6^ GW = 99.67%; PRN: percent decay <24 GW = 96.37%, 24–27^+6^ GW = 95.97%, 28–31^+6^ GW = 96.95%; TT: <24 GW =99.56%, 24–27^+6^ GW = 99.70%, 28–31^+6^ GW = 99.75%; DT: <24 GW = 97.25%, 24–27^+6^ GW = 98.45%, 28–31^+6^ GW = 98.70%).

A high number of breastmilk samples at 14 days had no quantifiable IgA: 69.1% (67/97) for PT, 48.5% (47/97) for TT and 16.5% (16/97) for DT. All breastmilk samples had quantifiable PRN-specific IgA.

## DISCUSSION

To our knowledge, this is the first time that the impact of the timing of pertussis vaccination in pregnancy has been explored in relation to antibodies in breastmilk. We found similar concentrations of PT, PRN, TT, or DT specific-IgA in colostrum or breastmilk at 14 days in women who had received vaccination in different time windows, all within the period recommended for vaccination in the UK.

Human breastmilk is a complex and dynamic source of nutrition that plays an important immunological role, particularly in the first few months of life as infants are developing their adaptive immunity.^20^ Breastfeeding has been established as protective for infants in preventing various respiratory infections;^21^ however, there is disagreement in the literature looking at breastfeeding and clinical pertussis cases. Exclusive breastfeeding was not a protective factor for pertussis in hospitalized infants under 6 months,^22^ and the incidence and prevalence of breastfeeding at the time of hospital admission were not different between pertussis-like cases and controls.^23^ In contrast, a study shows that breastfed infants had decreased pertussis odds compared to infants receiving more formula,^24^ and another study states that breastfeeding had a protective effect against pertussis in infants from both unvaccinated and vaccinated mothers.^25^

IgA is the dominant antibody found in breastmilk and supports the developing mucosal immunity by lining mucosal surfaces, including the respiratory tract.^26–28^ The significance of this in mediating protection against disease is uncertain, but it can be hypothesized that an increase in antibody concentration could result in increased protection for infants. Antenatal vaccinations have been shown to help increasing breastmilk IgA concentration for pertussis,^15,16^ pneumococcus,^29–31^ meningococcus,^32,33^ influenza A^34^ and SARS-CoV-2.^35^

Our finding that the timing of administration of a pertussis-containing vaccine in pregnancy did not result in any difference in PT and PRN-specific-IgA concentrations in breastmilk is interesting. Similarly, in the primary analysis of the OpTIMUM trial,^36^ we have shown equivalence of PT and PRN-specific IgG concentration in the cord blood of term infant at birth, regardless of the timing of antenatal vaccination. Equally, although there is no serocorrelate of protection for pertussis, recent effectiveness data from the UK show that there is similar effectiveness against pertussis in infants born to mothers vaccinated in both the second and third trimesters.^37^

Our study showed that specific-IgA concentrations were higher in colostrum than in 14 days breastmilk for all antigens. Similarly, multiple studies report that vaccine-specific IgA were higher in colostrum and lower in breastmilk at subsequent time points.^15–17,38^ Notably, a study comparing the colostrum and breastmilk at 2, 4 and 8 weeks postpartum, of unvaccinated and Tdap-vaccinated women after 20 weeks of gestation, found a significant decline in PT-specific IgA after 2 weeks in both groups.^16^ This is also consistent with the fact that total IgA levels are known to be higher in colostrum than in breastmilk.^39^

It is interesting that a high number of participants had IgA concentrations below the lower limit of detection for PT (69.1%) in transitional breastmilk at 14 days. A similar finding was made in a study using an ELISA assay at 1:101 milk dilution as per manufacturer instructions.^16^ Our multiplex bead assay requires a similar coefficient of dilution at 1:100. Although there are lower rates of samples with concentrations below the lower limit of quantification in 2 other studies using ELISA assays,^15,17^ breastmilk was only diluted at 1:5 or 1:10. The highest coefficient of dilution in our assay and in study^16^ can explain the higher rate of samples below the limit of detection. We would recommend exploring a lower coefficient of dilution for transitional and mature breastmilk in contrast to colostrum while being mindful of potential matrix effects.

### Limitations

This analysis includes participants from 2 separate studies with different methodologies for the recruitment and vaccination of participants. In the MAMA study, participants were recruited postnatally, and their vaccinations had been given as part of routine care. This may have led to inaccuracy in information about the timing of vaccination, although if there was any uncertainty from participants, the details were confirmed from medical records. The laboratory analysis for samples from both trials was conducted in the same laboratory using the same assays. Because we combined the data from 2 trials, we are only able to include results that were collected in both trials, which limited our ability to report on all the planned objectives. We also did not have an unvaccinated control group with which to compare our results.

Although we had originally planned to collect samples from participants 6 weeks after delivery, there was significant disruption in collecting these samples because of restrictions that took place because of the COVID-19 pandemic, and there were limited samples available at this time point, which made meaningful analysis impossible. This has limited our period of longitudinal assessment, which is unfortunate, as this is a weakness in previous studies.^17,40^

Finally, this was a small study that was not powered to show a difference between the study groups, and the conclusions we are able to draw are therefore limited and require further investigation in a larger study.

## CONCLUSIONS

In this study looking at different timing of administration of a pertussis-containing vaccine in pregnancy, no differences were identified in the specific-IgA antibody concentration in colostrum or breastmilk at 14 days for PT, PRN, DT or TT. Further work is required to investigate the relationship between the timing of vaccination and breastmilk protection. We suggest a study of larger scale with an unvaccinated control group, looking at breastfeeding, vaccination and clinical outcomes, different immune components present in milk including antibodies, and their functionality such as bactericidal activity and opsonophagocytosis.

### ACKNOWLEDGMENTS

*We are very grateful to the participants of the MAMA and OpTIMUM trials.*

*MAMA/OpTIMUM breastmilk study group members are:*

*Agnieszka Burtt, Emily Cornish, Danielle Hake, Tom Hall, Uzma Khan, Nicki Martin, Robin Parsons, Laura Sparks, Fiona Walbridge, Susan J. Wellstead and Myles Loughnan.*

## Supplementary Material


