# Supplementary material for: Antibody in Breastmilk Following Pertussis Vaccination in Three-time Windows in Pregnancy
Source: Pediatr Infect Dis J. 2025 Feb 14;44(2):S66–9. doi: 10.1097/INF.0000000000004696 (PMC12178168; doi:10.1097/INF.0000000000004696)

SUPPLEMENTAL DIGITAL CONTENT 1. Consort flow diagram. Participant recruitment, visits and sample collection for the MAMA study and the OpTIMUM study.

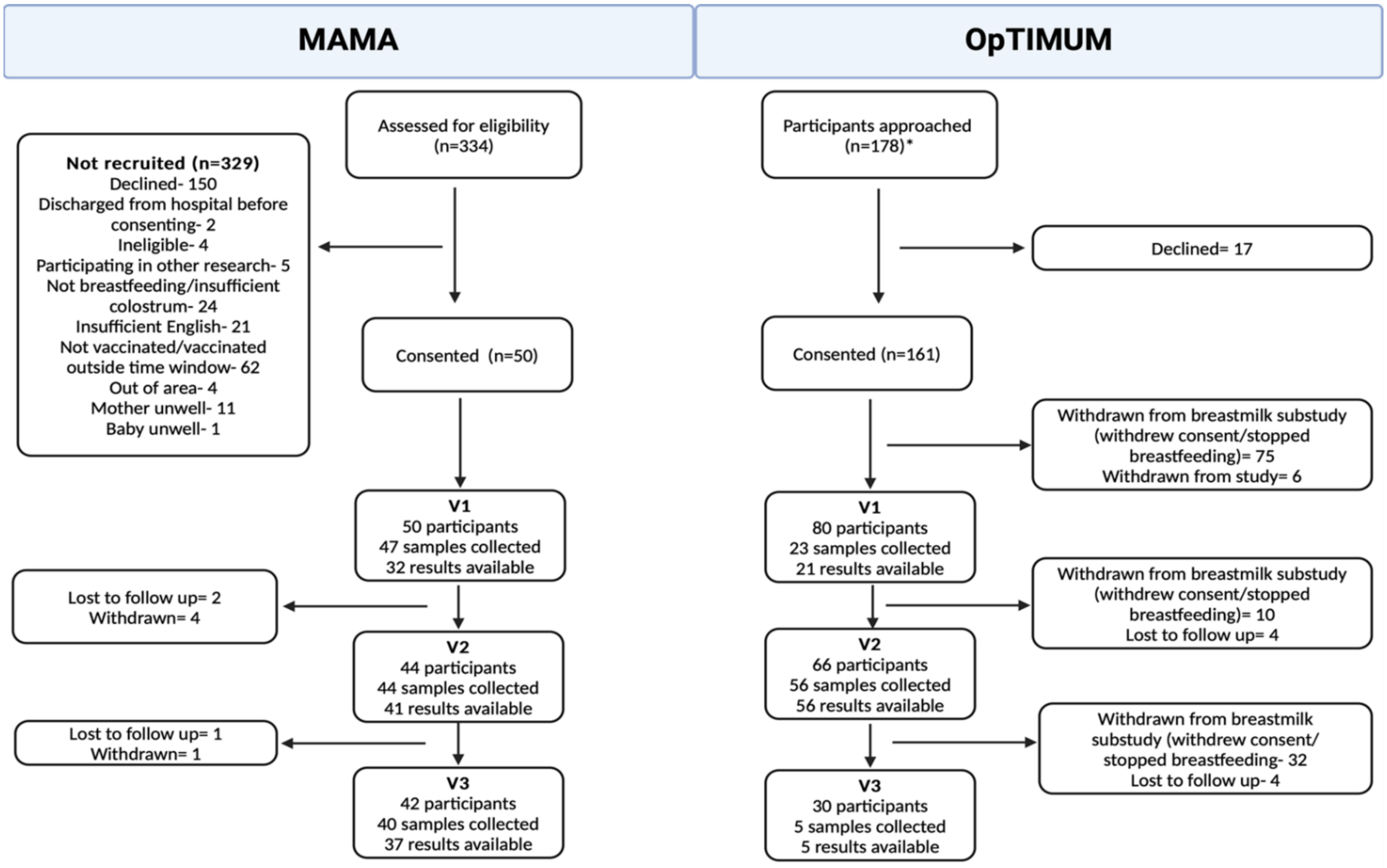

Supplement: Supplementary file 1 [file inf-44-s066-s001.pdf]
