# Supplementary material for: Antibody in Breastmilk Following Pertussis Vaccination in Three-time Windows in Pregnancy
Source: Pediatr Infect Dis J. 2025 Feb 14;44(2):S66–9. doi: 10.1097/INF.0000000000004696 (PMC12178168; doi:10.1097/INF.0000000000004696)
Supplement: Supplementary file 2 [file inf-44-s066-s002.pdf]

SUPPLEMENTAL DIGITAL CONTENT 2. Demographic details about participants who received vaccination in the three gestational age windows.

	**Group 1 (<24 GW) (n= 41)**	**Group 2 (24-27^{+6} GW) (n=40)**	**Group 3 (28-31^{+6} GW) (n=23)**
Median gestational age at vaccination (IQR)	21^{+5} (17^{+0}-23^{+6})	$24^{+6.5}$ (24^{+0}-27^{+6})	28^{+4} (28^{+0}-31^{+6})
Pertussis vaccination in a previous pregnancy (%)	19/41 (46.3)	23/40 (57.5)	12/23 (52.2)
White ethnicity (%)	33/41 (80.5)	35/40 (87.5)	21/23 (91.3)
Median gestational age at delivery (IQR)	40 (37^{+6}-41^{+6})	40^{+2} (37^{+2}-42^{+1})	40^{+1} (38^{+4}-41^{+6})

IQR= interquartile range; GW= gestational weeks.
