# Supplementary material for: Antibody in Breastmilk Following Pertussis Vaccination in Three-time Windows in Pregnancy
Source: Pediatr Infect Dis J. 2025 Feb 14;44(2):S66–9. doi: 10.1097/INF.0000000000004696 (PMC12178168; doi:10.1097/INF.0000000000004696)

SUPPLEMENTAL DIGITAL CONTENT 4. GMC of IgA against Pertussis toxin (A), Pertactin (B), Tetanus toxoid (C) and Diphtheria toxoid (D) in breastmilk at 14 days according to gestational window at vaccination. Geometric mean concentration and 95% confidence interval of antigen-specific IgA (AU). <24 GW (n=37), 24-27 GW (n=37), 28-31 GW (n=23). GW= gestational weeks.

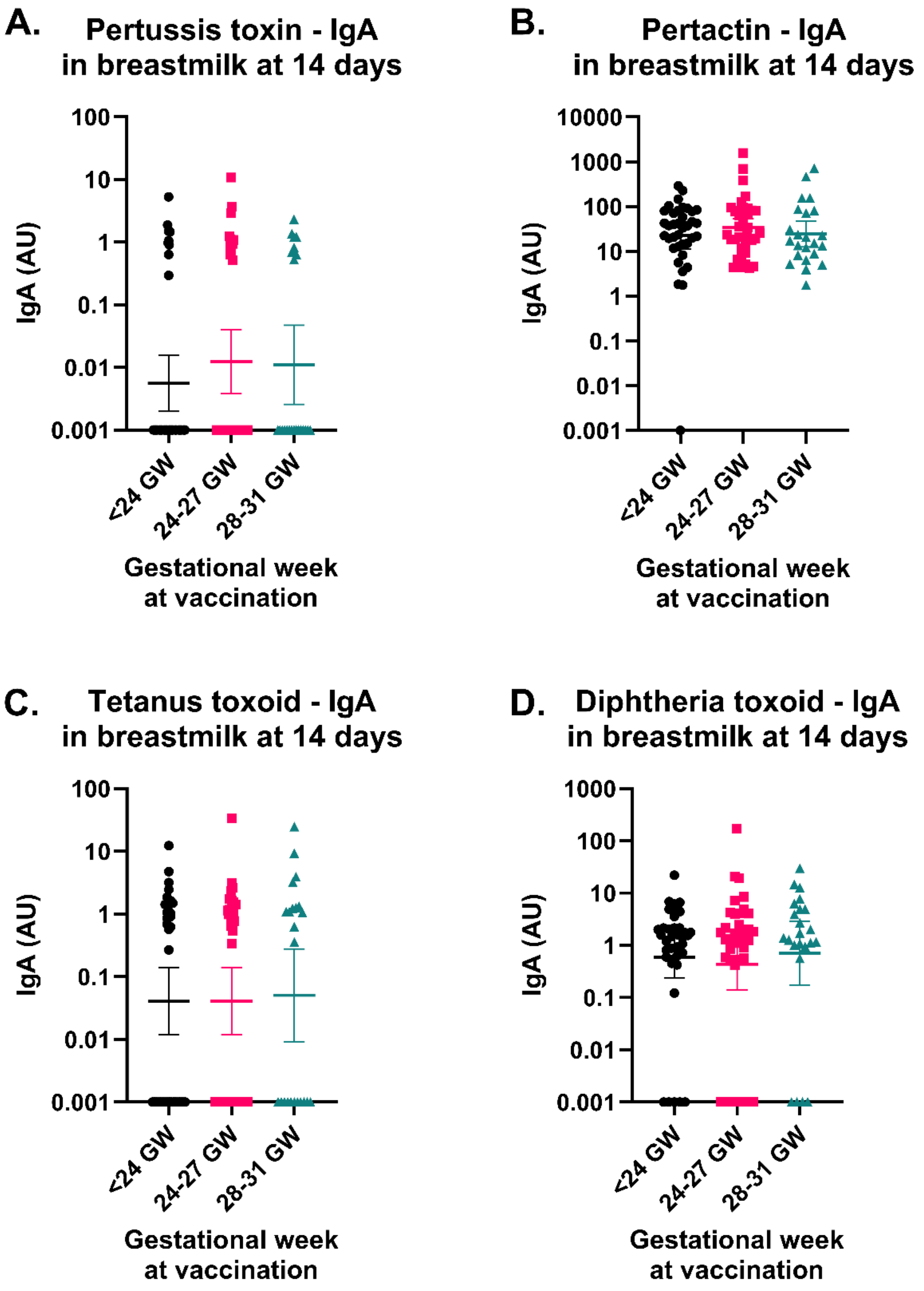

Supplement: Supplementary file 4 [file inf-44-s066-s004.pdf]
