# Supplementary material for: Antibody in Breastmilk Following Pertussis Vaccination in Three-time Windows in Pregnancy
Source: Pediatr Infect Dis J. 2025 Feb 14;44(2):S66–9. doi: 10.1097/INF.0000000000004696 (PMC12178168; doi:10.1097/INF.0000000000004696)

SUPPLEMENTAL DIGITAL CONTENT 5. Colostrum IgA and cord serum IgG, specific to Pertussis toxin (PT, A) and to Pertactin (PRN, B). Cord serum was collected at delivery and colostrum within 48 hours after delivery. For PT n=21 participants, for PRN n=19 participants.

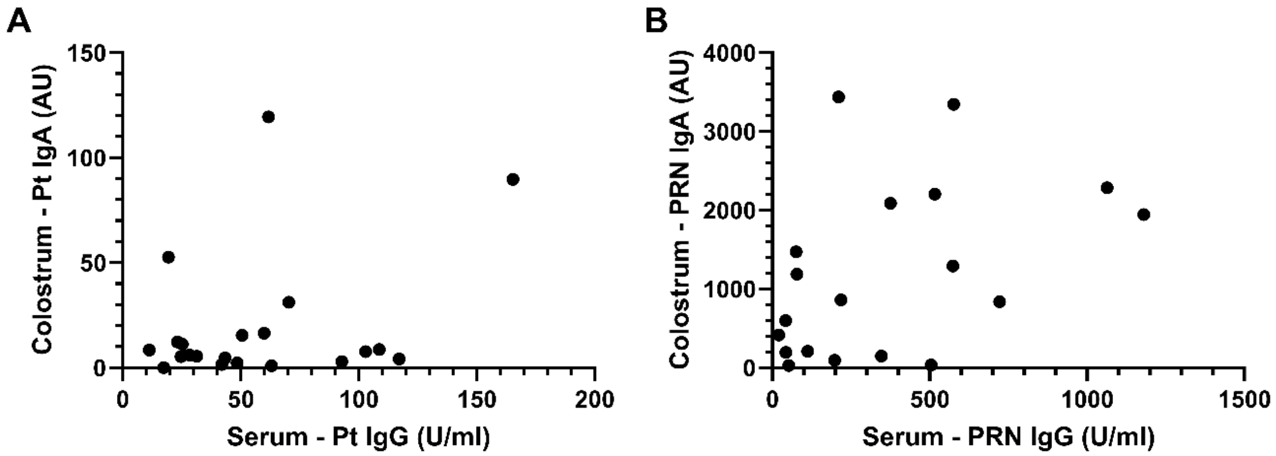

Supplement: Supplementary file 5 [file inf-44-s066-s005.pdf]
